# Validation of the Levenson Self-Report Psychopathy (LSRP) scale in the non-institutionalized Lebanese population

**DOI:** 10.1186/s12888-024-05499-4

**Published:** 2024-01-24

**Authors:** Elias Ghossoub, Hala Itani, Rayah Touma Sawaya, Pia Maria Ghanime, Michele Cherro, Martine Elbejjani, Marc Barakat, Khalil El Asmar

**Affiliations:** 1 Faculty of Medicine, American University of Beirut, Bliss Street, Beirut, Lebanon; 2 Boston University School of Public Health, 715 Albany Street, Boston, MA United States of America; 3 Massachusetts General Hospital, 55 Fruit Street, Boston Massachusetts, United States of America; 4 Faculty of Health Sciences, American University of Beirut, Bliss Street, P.O. Box 11-0236, Riad El-Solh/Beirut 1107 2020 Beirut, Lebanon

**Keywords:** Psychopathy, Lebanon, Factor analysis, Arab World

## Abstract

**Background:**

Psychopathy has been described as “the first personality disorder to be recognized in psychiatry”. It has three core features: affective, interpersonal, and behavioral. The Levenson Self-Report Psychopathy (LSRP) scale is used to screen for and measure psychopathy. Our study aims to validate the LSRP as a tool to measure psychopathy in the non-institutionalized Lebanese population.

**Methods:**

We surveyed Lebanese individuals residing in Lebanon and aged 18 through 65. It was a convenience sample collected via an online survey. 534 Lebanese participants completed the survey and were included in our analyses. Nearly 80% were female, 90% were college educated, and 60% were employed. We used exploratory graph analysis and confirmatory factor analyses to measure internal validity of the LSRP. We also used the HEXACO Personality Inventory-Revised (HEXACO-PI-R), the Subtypes of Antisocial Behavior Questionnaire (STAB), and the Short version of the Urgency, Premeditation (lack of), Perseverance (lack of), Sensation Seeking, Positive Urgency, Impulsive Behavior Scale (S-UPPS-P) to measure external validity of LSRP.

**Results:**

The exploratory graph analysis showed that the LSRP had a three-factor structure (Egocentric, Callous and Antisocial) in the Lebanese population. This three-factor structure (RMSEA = 0.05, CFI = 0.83, SRMR = 0.06) yielded a better fit than the two-factor, and three-factor Brinkley models. The LSRP was negatively correlated with the Honesty-Humility dimension of the HEXACO-PI-R and positively correlated with the STAB and S-UPPS-P subscales.

**Conclusions:**

The LSRP scale is a valid measure of psychopathy in the Lebanese non-institutionalized population, adding to the currently limited literature addressing psychopathy in the Arab World.

**Supplementary Information:**

The online version contains supplementary material available at 10.1186/s12888-024-05499-4.

## Background

Psychopathy was described as “the first personality disorder to be recognized in psychiatry” [1]. Decades of clinical and empirical investigation have yielded support for the validity of the concept of psychopathy, despite persistent controversy regarding the ‘concept’s boundaries as a construct [2, 3]. There is consensus that psychopathy has three core features: affective, interpersonal, and behavioral. Affective characteristics include callousness and lack of empathy. Interpersonal attributes comprise shallowness, grandiosity, pathological lying, and manipulation of others. The behavioral component of psychopathy mostly involves rule-breaking and impulsive tendencies [2, 4]. Psychopathy is believed to be the product of genetic and biological factors coupled with environmental and social forces; it usually manifests during juvenile years and progresses into adulthood [2].

Psychopathy is a strong predictor of future dangerousness and criminal recidivism [5–7]. Not surprisingly, while the prevalence of psychopathy is estimated at 1% in the general non-institutionalized population [4], it may reach 3% in forensic psychiatric samples [8] and 25% in incarcerated samples [2]. Additionally, psychopathy is correlated with impulsivity [9–11] and negatively correlated with personality traits such as honesty, humility, agreeableness, and conscientiousness [12–14].

Multiple tools were designed to screen for and measure the construct of psychopathy within the community and forensic populations. The Psychopathy Checklist–Revised (PCL-R) is considered the gold standard assessment tool [4]. It is a scale that relies on a semi-structured interview and a collateral and legal information review of the respondent [2]. Later on, other tools to measure psychopathy were developed and validated in multiple cultural contexts. One of the self-report measures of psychopathy is the 26-item LSRP scale, which reflects a dual-factor model of psychopathy: primary (interpersonal/affective features) and secondary (antisocial/lifestyle features) [15, 16]. LSRP was validated in North America and Western Europe [17]. However, some studies have suggested alternative factor structures for the LSRP, including a three-factor structure (egocentric, callous, and antisocial) [18] and a four-factor structure (deceitful/manipulative, superficial/selfish, callous, and antisocial) [19]. Brinkley’s three-factor model is the most commonly accepted LSRP model in the literature and validated in different countries [20–22]. However, it is important to consider cultural differences when evaluating psychopathy, as cultural attitudes, values, and beliefs can modulate an individual’s perception and interaction with their surroundings [17, 23].

Therefore, evaluating psychopathy in different socio-cultural contexts leads to a more comprehensive conceptualization of the disorder and its manifestation in different settings and provides insight into the pathophysiology and avenues for treatment.

There is limited data on the validity of the construct of psychopathy in the Arab culture. Some studies have found evidence supporting the validity of the construct in Arab-speaking countries, such as Egypt and Saudi Arabia [24, 25]. However, a study of Lebanese university students using the Psychopathic Personality Inventory-Revised raised questions about the validity of the psychopathy construct in the Arab culture due to questionable internal consistency of the scale [26].

Given the limited data and the need for a validated and reliable scale to measure psychopathy in the non-institutionalized Lebanese population, our study aimed to validate the LSRP as a tool to assess psychopathy in this population. Through exploratory graph analysis and confirmatory factor analysis, we identified our sample’s best model fit for the LSRP. We also assessed the scale’s internal and external validity by examining its correlations with personality traits, antisocial behaviors, and impulsivity measures.

## Methods

### Aim, design and setting of the study

The aim of this study is to validate the LSRP as a tool to assess psychopathy in the non-institutionalized Lebanese population. We recruited participants through online advertising on social media platforms such as Facebook, Twitter, and Instagram.

### Participants

We included participants who were Lebanese, residing in Lebanon, aged 18 through 65. We excluded non-Lebanese and Lebanese nationals living abroad.

### Procedure

Our study was approved by the Institutional Review Board (IRB) of the American University of Beirut (ID SBS-2020-0491). Since our research pertained to potentially sensitive information related to participants’ past experiences, our survey was anonymous and did not include private health information. Between May and June 2021, we invited participants to fill an online self-administered questionnaire via LimeSurvey, an online survey application, via convenience sampling. We informed participants about the study, its purpose, and inclusion criteria. Those willing to participate were required to read and accept an online consent form.

There is no gold-standard formula to calculate an adequate sample size for a factor analysis. Generally, the more robust the data, the smaller the sample size needed. The robustness is indicated by high communalities, no cross-loadings, strong loadings per factor, number of factors and number of items per factor [27]. Given the literature regarding the validity of the concept of psychopathy and the LSRP in the Lebanese population is very limited, a more cautious approach would be to aim for a large sample size. Based on Comrey and Lee’s work, we targeted a sample size of 500 participants [28]. 

### Measures

We used two versions in English and Arabic languages for each scale. We employed the back-translation method to ensure the linguistic and conceptual equivalence of the scales in Arabic. Two independent bilingual translators conducted the forward translation, and then two different bilingual translators performed the back translation. We reconciled any discrepancies through consensus discussions to ensure the accuracy and cultural relevance of the translations.

### Socio-demographic characteristics

Those include age, gender, marital status, educational level, occupation and area of residence.

### Levenson Self-Report Psychopathy scale (LSRP)

The LSRP is a 26-item scale designed to measure primary (affective and interpersonal dimensions) and secondary (behavioral dimension) psychopathy [16]. It was built based on the hypothesis that the construct of psychopathy is dimensional rather than categorical, with the understanding that psychopathic traits can be measured and detected in the general non-institutionalized population; individuals who are labeled “psychopaths” in a forensic setting exhibit a large number of those traits [16]. The LSRP has been validated in community settings [16, 21, 29] and in forensic settings [18]. We translated the LSRP to Arabic and back-translated it.

### HEXACO Personality Inventory-Revised (HEXACO-PI-R, 100-item version)

The HEXACO-PI-R is a self-report instrument that assesses the six major dimensions of personality: Honesty-Humility, Emotionality, eXtraversion, Agreeableness (versus Anger), Conscientiousness and Openness to Experience [30]. The HEXACO-PI-R consists of 100 items and is validated in the Lebanese population in English and the Omani population in Arabic [31]. We used the Arabic translation in the Omani study and culturally adapted some of its items.

### Subtypes of Antisocial Behavior Questionnaire (STAB)

The STAB is a 32-item self-report questionnaire validated to measure three different types of antisocial behavior: physical aggression, rule-breaking and social aggression [32]. The STAB is not available in the Arabic language. We translated it to Arabic and then back-translated it.

### Short version (20 items) of the urgency, Premeditation (lack of), perseverance (lack of), sensation seeking, positive urgency, impulsive behavior scale (S-UPPS-P)

The S-UPPS-P is a 20-item self-report questionnaire designed to measure the following five facets of impulsivity: positive urgency, negative urgency, lack of premeditation, lack of perseverance and sensation seeking. We used the English and Arabic versions which have been validated in Lebanon [33].

### Statistical analysis

An exploratory graph analysis (EGA) was conducted to explore the possible number of factors over which the measured items would load. EGA estimates the dimensionality structure of multivariate data by combining network analysis with a community detection algorithm (i.e., a method to detect dimensions in networks) [34] EGA is a dimensionality technique from the network psychometrics perspective. In Exploratory Graph Analysis (EGA), variables are visualized as nodes shaped like circles within a network, and the relationships among these variables are indicated by lines known as edges. The essence of the EGA algorithm involves constructing this network to discern patterns of connectivity. Specifically, it utilizes the Walktrap community detection algorithm, a method well-suited for spotting clusters of closely interlinked nodes within weighted networks [35]. In the network representation utilized for EGA, communities within the graph, which correspond to densely interconnected nodes, have been found to be statistically analogous to latent factors in factor analysis models. Prior to the application of EGA, we conducted a unique variable analysis (UVA) to address any potential redundancy among the items [34, 36]. We then used Confirmatory factor analysis as per the guidelines set forth in Brown (2006) to test the fit and adequacy of the model that was identified in EGA in addition to the Brinkley three-factor model [37]. We used the following global fit indices to assess the goodness of fit of the hypothesized factor structure and fit of the model: (i) Chi-square (χ2) statistic, (ii) root mean squared error of approximation (RMSEA), the standardized root mean square residual (SRMR), and the Comparative Fit Index (CFI) [38].

We calculated the Cronbach Alpha to assess the internal consistency of each of the proposed factors in our 3 factors model. We analyzed the data using R version 4.3.2 [EGAnet and psychTools] [39–41] and Stata version 18 [28]. The R and Stata code we used in our analyses is available in Supplementary Material 3.

## Results

### Sample characteristics

A total of 1541 individuals clicked on the link to access the survey, out of which 572 participants filled the survey. We included 534 Lebanese participants who completed at least 80% of items of all the scales. Out of the total sample, 78.1% were female. More than half of our sample were single. Around 90% of our sample were college educated, 61% were employed and 78% were living in a city. Our included sample’s characteristics were similar to those we excluded due to missing data, except for age: included participants had a statistically significant younger age that those excluded (*p* = 0.022) (Supplementary Material 1). Further details are available in Table 1.

**Table 1:** Socio-Demographic Characteristics of the Included Sample

Characteristic	Participants (*N* = 534)	
	Mean	Standard Deviation
** Age**	36.6	11.9
	**N**	**Percent**
**Gender**		
** Male**	110	20.6%
** Female**	417	78.1%
** Non-binary**	4	0.7%
** Not listed**	2	0.4%
** Transgender male**	1	0.2%
**Nationality**		
** Lebanese**	534	100.0%
**Current residence**		
** Lebanon**	534	100.0%
**Marital status**		
** Married**	230	43.1%
** Divorced**	23	4.3%
** Separated**	5	0.9%
** Single**	274	51.3%
** Widowed**	2	0.4%
**Education Level**		
** Less than high school**	5	0.9%
** High school**	28	5.2%
** Technical school**	14	2.6%
** University**	487	91.2%
**Employment status**		
** Employed**	323	60.5%
** Homemaker**	17	3.2%
** Retired**	23	4.3%
** Student**	87	16.3%
** Unemployed**	84	15.7%
**Location**		
** Rural (countryside)**	118	22.1%
** Urban (city)**	416	77.9%

### Exploratory graph analysis

**Fig. 1:**
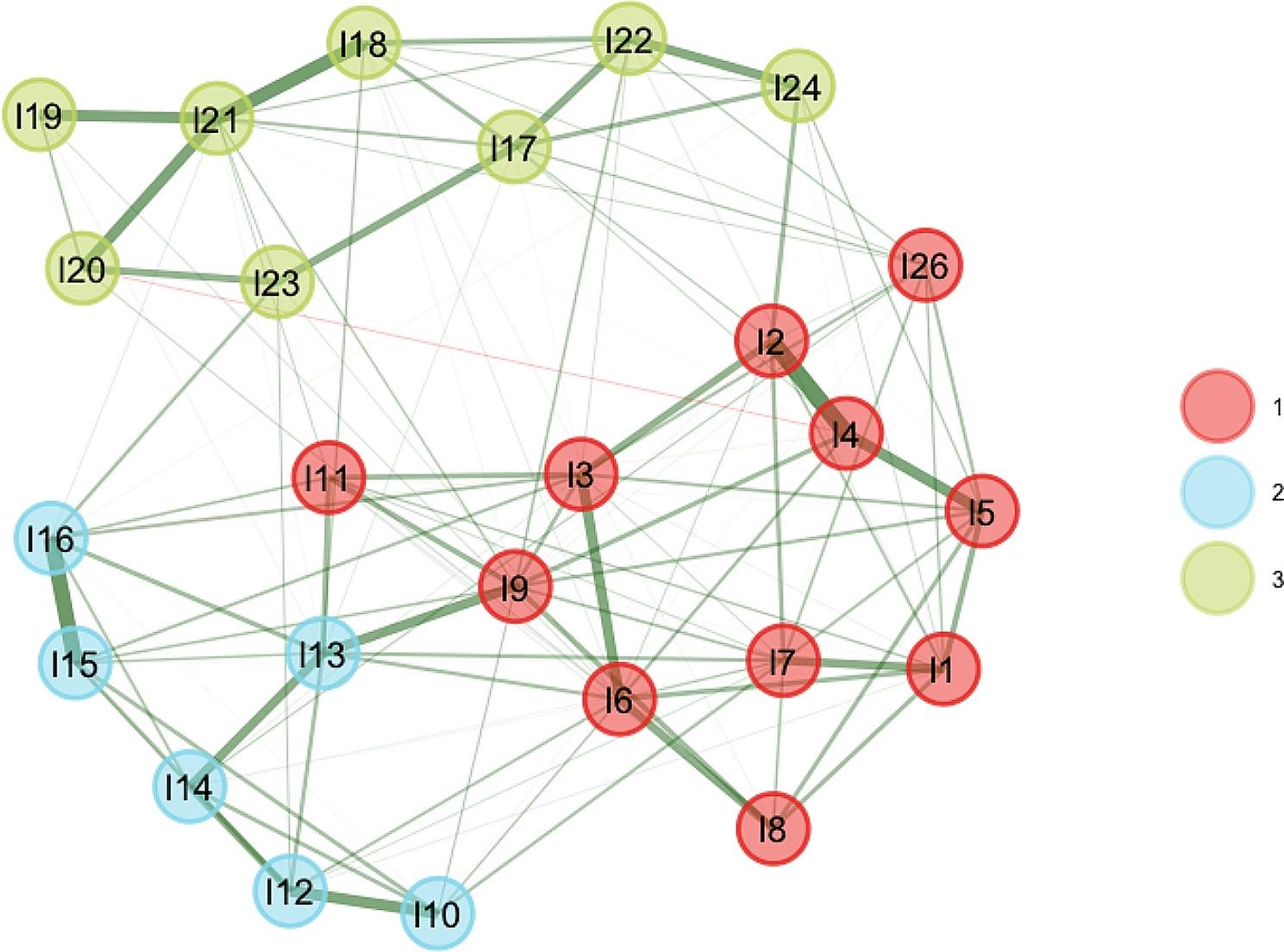
Exploratory Graph Analysis Depicting the Three-Factor Structure of the LSRP

Results from the EGA (Fig. 1) revealed a three-factor structure: Egocentric (11 items), Callous (6 items) and Antisocial (8 items). The pair items 24 and 25 exhibited large to very large redundancies in the EGA analysis; as a result we excluded item 25 from the analysis. The three-factors structure exhibited strong dimensions stability (Supplementary Material 2) and a low Total Entropy Fit Index (TEFI) of -21.297.

### Confirmatory factor analysis

Table 2 shows the results of the scaled model χ^2^, RMSEA, CFI, SRMR, Akaike information criterion (AIC) and Bayesian Information Criterion (BIC). The three-factor model yielded a better fit in terms of the values of the RMSEA, SRMR and CFI than the two-factor, and the Brinkley models (RMSEA = 0.05, CFI = 0.83, SRMR = 0.06 for the three-factor model; RMSEA = 0.072, CFI = 0.72, SRMR = 0.07 for the two-factor model; RMSEA = 0.076, CFI = 0.782, SRMR = 0.06 for the Brinkley model). Although the initial three-factor model had a better fit than the other proposed models, we still produced the modification indices and respecified a model which allowed the following two items to have residuals correlated because of conceptual similarities: (i) “For me, what’s right is whatever I can get away with” and (ii) “My main purpose in life is getting as many goodies as I can”. The fit indices improved from the initially specified three-factor model as follows: ΔX2[df = 1]: -70.3, ΔCFI:+0.03, ΔSRMR:-0.03.

**Table 2:** Summary of Fit Statistics for LSRP Models

	Two-Factor-Model	Three-Factor-Model	Brinkley Model
χ^2^ (*p*-value)	< 0.001	< 0.001	< 0.001
RMSEA (95% CI)	0.07 (0.07–0.08)	0.05 (0.05–0.06)	0.07 (0.07–0.08)
CFI	0.71	0.83	0.78
SRMR	0.07	0.06	0.07
AIC	27,814	26,958	20,653
BIC	28,147	27,293	20,908

LSRP: Levenson Self-Report Psychopathy Scale; RMSEA: Root Mean Square Error Of Approximation; CFI: Comparative Fit Index; SRMR: Standardized Root Mean Squared Residual; AIC: Akaike Information Criterion; BIC: Bayesian Information Criterion

We extracted factor scores for the respecified three-factor model. Table 3 displays the correlations between latent factors as follows: 0.47 (Callous and Egocentric), 0.41 (Antisocial and Egocentric), 0.25 (Antisocial and Callous). All correlations were significant with a *p*-value < 0.001. Cronbach’s α for the three factors were 0.80 for Egocentric, 0.68 for Callous and 0.67 for Antisocial.

**Table 3:** Correlation Matrix for the Three Latent Factors of the LSRP

Factors	Egocentric	Callous	Antisocial
**Egocentric**	-		
**Callous**	0.47*	-	
**Antisocial**	0.41*	0.25*	-

LSRP: Levenson Self-Report Psychopathy Scale

*p < 0.001

### Correlation of LSRP with other scales

Examining the correlation of both the total scores and identified factors of the LRSP, we found that the LSRP had negative correlations with several dimensions of the HEXACO-PI-R. It had the strongest negative correlation with the Honesty-Humility dimension (*r*=-0.6022). Furthermore, the Egocentric and Callous factors had the strongest negative correlation with the Honesty-Humility dimension (*r*=-0.5683 and *r*=-0.4962 respectively) while theAntisocial factor had the strongest negative correlation with the Conscientiousness dimension (*r*=-0.4949).

We measured the correlation of the total LSRP score with the individual dimensions of the STAB and found that it had the strongest positive correlation with social aggression (*r* = 0.4951). We also measured the correlation of the LSRP’s three factors with the STAB’s three dimensions. Both the Callous factor and the Egocentric factor had the strongest positive correlations with social aggression (*r* = 0.3570 *r* = 0.3883 respectively) while Antisocial factor had the strongest positive correlation with physical aggression (*r* = 0.4477).

Correlating LSRP to SUPPS-P, the total LSRP score had the strongest positive correlation with positive urgency (*r* = 0.4045). The Egocentric factor had the strongest positive correlation with positive urgency (*r* = 0.3498). The Callous factor had the strongest positive correlation with lack of perseverance (*r* = 0.1630). Finally, the Antisocial factor had the strongest positive correlation with negative urgency (*r* = 0.4220). Table 4 shows correlation matrix between LSRP factors and HEXACO scale, SUPPS-P and STAB scale.

**Table 4:** Correlation Matrix between LSRP and Other Scales

Scale	Factors	Egocentric	Callous	Antisocial	Total
**HEXACO-PI-R**	**Honesty-Humility**	-0.5683**	-0.4962**	-0.2824**	-0.6022**
	**Emotionality**	-0.2177**	-0.2278**	0.0665	-0.1714**
	**Extraversion**	-0.1122**	-0.1065**	-0.3539**	-0.2420**
	**Agreeableness**	-0.2784**	-0.2200**	-0.3050**	-0.3554**
	**Conscientiousness**	-0.1722**	-0.2662**	-0.4949**	-0.3828**
	**Openness to experience**	-0.2655**	-0.1224**	-0.1612**	-0.2601**
**STAB**	**Physical Aggression**	0.3592**	0.2737**	0.4477**	0.4774**
	**Social Aggression**	0.3883**	0.3570**	0.3865**	0.4951**
	**Rule-Breaking**	0.3174**	0.2620**	0.2814**	0.3817**
**S-UPPS-P**	**Positive Urgency (Total)**	0.3498**	0.1357**	0.3842**	0.4045**
	**Negative Urgency (Total)**	0.2606**	0.0722	0.4220**	0.3475**
	**Lack of Perseverance (Total)**	0.1069*	0.1232**	0.3712**	0.2509**
	**Lack of Premeditation (Total)**	0.2090**	0.1455**	0.3698**	0.3089**
	**Sensation Seeking (Total)**	0.2210**	0.1630**	0.1555**	0.2365**

LSRP: Levenson Self-Report Psychopathy Scale; HEXACO-PI-R: Honesty-Humility, Emotionality, eXtraversion, Agreeableness (versus Anger), Conscientiousness and Openness to Experience Personality Inventory-Revised; STAB: Subtypes of Antisocial Behavior Questionnaire; S-UPPS-P: Short version of the Urgency, Premeditation (lack of), Perseverance (lack of), Sensation Seeking, Positive Urgency, Impulsive Behavior Scale

*p < 0.05

**p < 0.01

## Discussion

The present study aimed to validate the LSRP as a tool to measure psychopathy in the non-institutionalized Lebanese population. We assessed the scale’s internal and external validity by identifying correlations between the LSRP and measures of personality traits (HEXACO-PI-R), antisocial behaviors (STAB), and impulsivity (SUPPS-P). Our factor analysis yielded a three-factor, 25-item model (Egocentric, Callous and Antisocial) as the best fit compared to the original two-factor model (primary psychopathy and secondary psychopathy) and the Brinkley three-factor, 19-item model. Although older studies [42, 43] replicated the original two-factor model [16], more recent studies (19–21, 44–45) successfully replicated the Brinkley three-factor model [18] using confirmatory factor analysis. Other studies [44, 45] also replicated a three-factor model, with several differences to the Brinkley model [18]. Popov et al. (2015) validated a Bulgarian version of the LSRP on a sample of 379 participants: the authors reported that while Brinkley’s model had an acceptable fit, exploratory factor analyses found that a four-factor model (deceitful/manipulative, superficial/materialistic, lack of empathy and irritable/impulsive) yielded a better fit [46]. Our analysis led us to conclude that our three-factor model emerged as the optimal fit, requiring only the removal of one item (item 25) out of the original 26 items. This finding underscores the robustness and applicability of our proposed model within the socio-cultural context of our study.

Brinkley et al. (2008) attempted to validate the LSRP in a sample of incarcerated women [18]. The authors didn’t find a good fit for the two-factor model of the original scale, therefore; they opted to perform an exploratory factor analysis. They concluded that the LSRP was formed by three factors: Egocentric (10 items), Callous (4 items), and Antisocial (5 items). Brinkley’s team removed all items with loadings less than 0.4, yielding a modified 19-item scale [18]. However, they did not carry out a confirmatory factor analysis. In our study, we conducted an exploratory graph analysis and a confirmatory factor analysis, validating a three-factor structure of the LSRP that performed better than the Brinkley model in our sample. Our study’s finding is similar to a validation study in Brazil [47], where the authors argued that structural concerns that were raised about the LSRP in samples in North American and/or Europe do not necessarily generalize to other cultural contexts, and that factor analyses can address cultural invariance of the tool.

The LSRP behaved as expected when correlated with measures of personality traits, antisocial behaviors, and impulsivity. We found that the LSRP had negative correlations with several dimensions of the HEXACO-PI-R. The total LSRP score had the strongest negative correlation with the Honesty-Humility dimension. More specifically, the Egocentric and Callous, factors had the strongest negative correlation with the Honesty-Humility dimension. Different scales for psychopathy have been used including self-reported scales [10]. Most studies involving self-reported scales use the Self-Report Psychopathy (SRP-III) scale and show that psychopathy was most strongly associated with low Honesty-Humility [12, 14]. Using the same SRP-III, Gaughan et al. (2012) found significant negative relations with the domains of Emotionality, Agreeableness, and Conscientiousness [13]. People who score low on Honesty-Humility tend to be involved in deceiving, cheating, and manipulative behaviors [48]. These behaviors align with the Egocentric and Callous factors in our model. The Antisocial factor had the strongest negative correlation with the Conscientiousness dimension. This is expected since people who score low on this dimension (and high on the Antisocial factor) tend to make impulsive decisions and are unconcerned with fulfilling tasks to a high standard [14].

Similarly, we measured the correlations of the LSRP with the STAB and found that it had strong positive correlations with all dimensions. Both the Egocentric and Callous factors had the strongest positive correlation with social aggression, while the Antisocial factor had the strongest positive correlation with physical aggression. This is expected as the social aggression dimension involves blaming others and hurting others’ feelings, and the physical aggression dimension involves direct forms of verbal and physical aggression [33]. Previous research has also found similar correlations found between psychopathy traits and antisocial behavior [16].

Moreover, several studies explored the association between psychopathy and impulsivity using the UPPS-P [11]. Interestingly, we found that LSRP factors correlated differently with different dimensions of the S-UPPS-P, albeit in the same (positive) direction. The Antisocial factor correlated strongly with positive and negative urgency which measure impulsivity due to emotion reactivity [49]. Prior research has shown that affective states may be linked with antisocial behaviors among psychopaths [50]. Moreover, the Antisocial factor had a strong positive correlation with lack of perseverance and lack of premeditation. This was expected as that factor includes items measuring the tendency to get bored, to quickly lose interest in tasks and to be short-sighted.The Egocentric factor also correlated strongly with positive and negative urgency. This association has been repeatedly demonstrated in the literature [51]. Indeed, egocentric people and narcissists engage in a pattern of self-enhancing and short-sighted behaviors, which are typical among impulsive individuals [51].

To our knowledge, this is the first study that validates a self-report measure of psychopathy in Lebanon and the Arab World, both in English and in Arabic, using a large sample size. Our study addresses a gap in forensic psychiatry research in Lebanon and the Arab region. Having correlates to psychopathy originating from well-known metrics establishes some characteristics of psychopathy of the Lebanese general adult population. In addition, some of the surveys were translated into Arabic and then back-translated into English. This step ameliorated the inclusivity of the surveys administered despite them not originating from Lebanon.

Our study also had some limitations. First, our sample had a higher proportion of women and English speakers. Therefore, we conducted sensitivity analysis on the male and Arabic language subsets. The three-factor model yielded a poor fit for both subsets. We decided to test whether the reason for this poor fit is due to small sample size or due to lack of validity among males and/or Arabic speakers. As a result we took a random sample of 150 respondents from the data set and tested the three-factor model. Model fit was also poor. We therefore concluded that the most likely reason for the poor fit for the males subsets and the Arabic language subsets is the small sample size rather than the sample characteristics. Second, we did not compare the performance of the LSRP to a validated gold-standard measure of psychopathy such as the PCL-R or its self-report version the SRP, since we are not aware that such tools have been validated in the Arab World. We note however that the LSRP demonstrated convergent validity with scales measuring personality traits, impulsivity, and antisocial behaviors in our sample. Third, our survey was conducted online. Although online surveys have several advantages (low cost, wider reach, convenience), they have some inconveniences: they are more likely to attract a highly educated population who have internet access, which may indicate selection bias [52]. Furthermore, our included sample’s mean age was significantly younger than those who were excluded for missing data. Fourth, the STAB was not validated in Arabic, but it primarily serves as a checklist of antisocial behaviors. This is a limitation to be considered in the interpretation of the study results. Fifth, we used an etic approach by validating a psychopathy scale originating from North America, as opposed to an emic approach that would require designing an indigenous culture-specific measurement tool of the construct. Researchers have argued that a combined etic-emic approach can be best in studying personality pathology cross-culturally [53].

The LSRP is a valid scale to measure psychopathy in the Lebanese non-institutionalized population. This is much needed given the limited research on psychopathy in the Arab world [54]. Future research is needed to validate the LSRP in forensic settings, to gain further insight into the epidemiology of psychopathy in Lebanon and the Arab world.

### Electronic supplementary material

Below is the link to the electronic supplementary material.

**Supplementary Material 1**: Characteristics of Survey Completers and Excluded Participants due to Missing Data

**Supplementary Material 2**: Stability of Item-Community Membership in Network Analysis

**Supplementary Material 3**: Stata and R Codes for Statistical Analysis

## Data Availability

The datasets used and analyzed during the current study are available from the corresponding author on reasonable request.

## Abbreviations

LSRP: Levenson Self-Report Psychopathy
HEXACO-PI-R: Honesty-Humility, Emotionality, eXtraversion, Agreeableness (versus Anger), Conscientiousness and Openness to Experience Personality Inventory-Revised
STAB: Subtypes of Antisocial Behavior Questionnaire
S-UPPS-P: Short version of the Urgency, Premeditation (lack of), Perseverance (lack of), Sensation Seeking, Positive Urgency, Impulsive Behavior Scale
PCL-R: Psychopathy Checklist–Revised
IRB: Institutional Review Board
EGA: Exploratory graph analysis
RMSEA: Root mean squared error of approximation
SRMR: Standardized root mean square residual
CFI: Comparative Fit Index
AIC: Akaike information criterion
BIC: Bayesian Information Criterion
SRP-III: Self-Report Psychopathy
