# Supplementary material for: Validation of the Levenson Self-Report Psychopathy (LSRP) scale in the non-institutionalized Lebanese population
Source: BMC Psychiatry. 2024 Jan 24;24:72. doi: 10.1186/s12888-024-05499-4 (PMC10809519; doi:10.1186/s12888-024-05499-4)
Supplement: Supplementary file 1 — Supplementary Material 1: Characteristics of Survey Completers and Excluded Participants due to Missing Data [file 12888_2024_5499_MOESM1_ESM.docx]

|  | COMPLETERS | | EXCLUDED | | P- VALUE |
| --- | --- | --- | --- | --- | --- |
|  | **N** | **pERCENT** | **N** | **PERCENT** |  |
|  | 534 | 93.4 | 38 | 6.6 |  |
| AGE (MEAN +/- STANDARD DEVIATION) | 36.6 ±11.9 | | 41.2 ±13.2 | | 0.022* |
| GENDER |  |  |  |  | 0.570 |
| MALE | 110 | 20.6 | 5 | 13.2 |  |
| FEMALE | 417 | 78.1 | 33 | 86.8 |  |
| NON-BINARY | 4 | 0.7 | 0 | 0 |  |
| NOT LISTED | 2 | 0.4 | 0 | 0 |  |
| TRANSGENDER MALE | 1 | 0.2 | 0 | 0 |  |
| MARITAL STATUS |  |  |  |  | 0.329 |
| MARRIED | 230 | 43.1 | 14 | 36.8 |  |
| DIVORCED | 23 | 4.3 | 2 | 5.3 |  |
| SEPARATED | 5 | 0.9 | 0 | 0 |  |
| SINGLE | 274 | 51.3 | 21 | 55.3 |  |
| WIDOWED | 2 | 0.4 | 1 | 2.6 |  |
| LEVEL OF EDUCATION |  |  |  |  | 0.526 |
| LESS THAN HIGH SCHOOL | 5 | 0.9 | 1 | 2.6 |  |
| HIGH SCHOOL | 28 | 5.2 | 2 | 5.3 |  |
| TECHNICAL SCHOOL | 14 | 2.6 | 0 | 0 |  |
| UNIVERSITY | 487 | 91.2 | 35 | 92.1 |  |
| EMPLOYMENT STATUS |  |  |  |  | 0.967 |
| EMPLOYED | 323 | 60.5 | 26 | 68.4 |  |
| HOMEMAKER | 17 | 3.2 | 1 | 2.6 |  |
| RETIRED | 23 | 4.3 | 1 | 2.6 |  |
| STUDENT | 87 | 16.3 | 5 | 13.2 |  |
| UNEMPLOYED | 84 | 15.7 | 5 | 13.2 |  |
| LOCATION |  |  |  |  | 0.840 |
| RURAL (COUNTRYSIDE) | 118 | 22.1 | 9 | 23.7 |  |
| URBAN (CITY) | 416 | 77.9 | 29 | 76.3 |  |

**Supplemental Table 1: Characteristics of Survey Completers and Excluded Participants due to Missing Data**

*p<0.05
