# Supplementary material for: Validation of the Levenson Self-Report Psychopathy (LSRP) scale in the non-institutionalized Lebanese population
Source: BMC Psychiatry. 2024 Jan 24;24:72. doi: 10.1186/s12888-024-05499-4 (PMC10809519; doi:10.1186/s12888-024-05499-4)

**Supplemental Figure 1: Stability of Item-Community Membership in Network Analysis**

This figure illustrates the stability of items' membership in identified communities through replication in a network analysis. Communities are denoted by colors (Red for Factor 1: Egocentric; Blue for Factor 2 Callous; Green for Factor 3: Antisocial). The numbers on the vertical axis indicate specific items of the Levenson Self-Report Psychopathy (LSRP) scale. The horizontal axis represents the replication proportion, with values closer to 1 indicating higher stability in community membership across bootstrap samples.
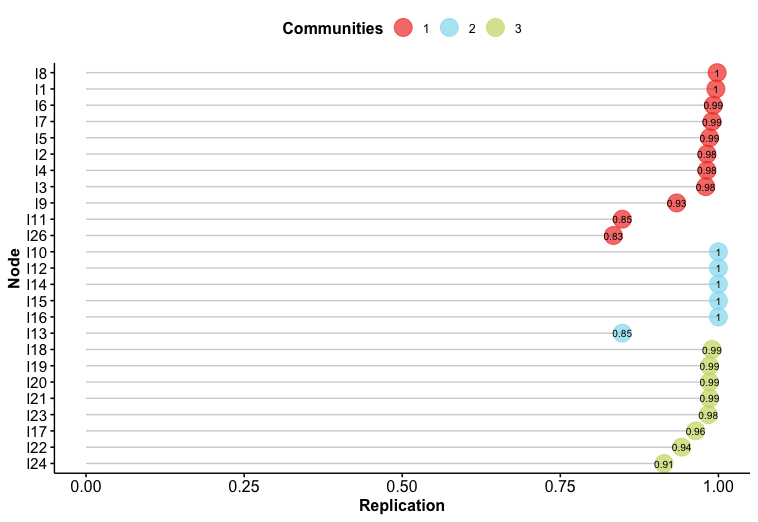

Supplement: Supplementary file 2 — Supplementary Material 2: Stability of Item-Community Membership in Network Analysis [file 12888_2024_5499_MOESM2_ESM.docx]
