# Supplementary material for: Validation of the Levenson Self-Report Psychopathy (LSRP) scale in the non-institutionalized Lebanese population
Source: BMC Psychiatry. 2024 Jan 24;24:72. doi: 10.1186/s12888-024-05499-4 (PMC10809519; doi:10.1186/s12888-024-05499-4)
Supplement: Supplementary file 3 — Supplementary Material 3: Stata and R Codes for Statistical Analysis [file 12888_2024_5499_MOESM3_ESM.docx]

**Supplement 1: Stata and R Codes for Statistical Analysis**

* Defining Lower-Order Factors

* Using the model as proposed by the EGA

sem (Egocentric -> l1-l9 l11 l26) (Callous -> l10 l12-l16) (Lifestyle -> l17-l25)

estat gof, stats(all) // Goodness-of-fit statistics

estat mindices // Modification indices

* Testing Model Excluding Items 24 and 25 due to Redundancy

sem (Egocentric -> l1-l9 l11 l26) (Callous -> l10 l12-l16) (Lifestyle -> l17-l23), covariance (e.l2*e.l4)

estat gof, stats(all) // Checking fit after item exclusion

* Testing Model Excluding Item 25 Only

sem (Egocentric -> l1-l9 l11 l26) (Callous -> l10 l12-l16) (Lifestyle -> l17-l24), covariance (e.l2*e.l4)

estat gof, stats(all) // Checking fit for this model variation

* Brinkley Model Analysis

sem (Egocentric ->l1-l7 l9 l11 l13) (Callous -> l12 l14 l15 l16) (Antisocial -> l17 l18 l21 l24 l25)

sem, standardized // Standardizing coefficients

estat gof, stats(all) // Goodness-of-fit for Brinkley model

* Testing a 2-Factor Model

sem (PS ->l1-l16) (SS -> l17-l26)

sem, standardized // Standardizing coefficients for the 2-factor model

* Creating Composite Scores for the 3 LSRP Factors

egen egocentric1 = rowtotal(l1-l9 l11 l26)

egen callous1 = rowtotal(l10 l12-l16)

egen lifestyle1 = rowtotal(l17-l24)

* Correlating HEXACO Domains with 4 Factors from LSRP

pwcorr Honesty_Humility Emotionality Extraversion Agreableness Conscientiousness Openness_Experience egocentric1 callous1 lifestyle1 LSRP_Total1, sig

graph matrix Honesty_Humility Emotionality Extraversion Agreableness Conscientiousness Openness_Experience egocentric callous lifestyle antisocial, half

* Correlating LSRP Factors with STAB and SUPPS Scales

pwcorr egocentric1 callous1 lifestyle1 LSRP_Total1 STAB_Physical_Total STAB_Social_Total STAB_Rules_Total, sig

pwcorr egocentric callous lifestyle antisocial LSRP_Total NegativeUrgency_Total LackPerseverance_Total LackPremeditation_Total SensationSeeking_Total PositiveUrgency_Total, sig

* Additional Correlations with STAB and SUPPS

pwcorr STAB_Physical_Total STAB_Social_Total STAB_Rules_Total PositiveUrgency_Total NegativeUrgency_Total LackPerseverance_Total LackPremeditation_Total SensationSeeking_Total egocentric1 callous1 lifestyle1 LSRP_Total1, sig

##Rcode####

# Ensure necessary packages are installed and loaded

if (!require("EGAnet")) install.packages("EGAnet")

library(EGAnet)

# Assuming LSRP_EGA is your dataset containing LSRP items

# Ensure that LSRP_EGA is a dataframe with appropriate item columns

# Perform Exploratory Graph Analysis (EGA)

LSRP_ega <- EGA(LSRP_EGA)

# Plot the EGA network

plot(LSRP_ega, layout = "spring")

# Perform Bootstrap EGA for stability assessment

set.seed(1) # Setting seed for reproducibility

LSRP_boot <- bootEGA(

data = LSRP_EGA,

nBoots = 1000, # Number of bootstrap samples

type = "nonparametric" # Type of bootstrap

)

# Compare Empirical and Bootstrap EGA plots

compare_EGA_plots <- compare.EGA.plots(

LSRP_ega, LSRP_boot,

labels = c("Empirical", "Bootstrap")

)

# Check dimension stability using the bootstrap results

dimension_stability <- dimensionStability(LSRP_boot)

# Apply the Louvain algorithm for community detection

# and assess the fit across different resolution parameters

fit_louvain <- EGA.fit(

data = LSRP_EGA, algorithm = "louvain",

resolution_parameter = seq.int(0, 2, 0.05),

plot.EGA = TRUE # Plotting the EGA for each resolution

)
